# Tirzepatide non-response with subsequent semaglutide response in class 2 obesity

**DOI:** 10.1210/jcemcr/luag245

**Published:** 2026-08-31

**Authors:** Sandro La Vignera, Rosita A Condorelli

**Affiliations:** Department of Clinical and Experimental Medicine, University of Catania, Catania 95123, Italy; Department of Clinical and Experimental Medicine, University of Catania, Catania 95123, Italy

**Keywords:** obesity, tirzepatide, semaglutide, GLP-1 receptor agonist, insulin resistance, precision medicine

## Abstract

We report a paradoxical case of a 40-year-old man with class 2 obesity (body mass index 39.8 kg/m^2^) and insulin resistance (homeostatic model assessment of insulin resistance [HOMA-IR] 5.0; reference <2.0) who achieved minimal weight loss (<5%) despite tirzepatide dose escalation to 15 mg weekly over 6 months. Following switch to semaglutide 2.4 mg weekly, the patient achieved 20% weight loss over 6 months, with normalization of fasting glucose, resolution of insulin resistance, and marked improvement in appetite control. This case challenges the assumption that dual glucose-dependent insulinotropic polypeptide (GIP)/glucagon-like peptide-1 (GLP-1) receptor agonism is universally superior to selective GLP-1 receptor agonism for weight management. Mechanistic analysis suggests that adipose tissue GIP receptor downregulation in the context of class 2 obesity and insulin resistance, combined with intact central GLP-1 receptor signaling, may explain the differential response in some individuals. Key learning points include: markedly elevated baseline insulin resistance (HOMA-IR ≥4-5) may be associated with poor tirzepatide response, though prospective validation is required; persistent hyperphagia during tirzepatide treatment is an early warning sign; and nonresponse to tirzepatide does not preclude semaglutide response. This case supports precision medicine approaches in obesity pharmacotherapy, including pretreatment phenotyping and early response assessment.

## Introduction

Tirzepatide, a dual glucose-dependent insulinotropic polypeptide (GIP) and glucagon-like peptide-1 (GLP-1) receptor agonist, has demonstrated superior average weight loss compared to semaglutide in randomized controlled trials [1, 2]. This has led to the widespread assumption that dual-agonist therapy is universally preferable for obesity management. However, population-level efficacy data may not reflect individual patient responses, particularly in phenotypically distinct subgroups. We present a case that challenges this assumption: a patient with class 2 obesity and marked insulin resistance who failed to respond to maximum-dose tirzepatide but achieved exceptional weight loss with semaglutide, a selective GLP-1 receptor agonist. This case highlights the critical role of metabolic phenotyping and early response assessment in guiding personalized obesity pharmacotherapy.

## Case presentation

A 40-year-old man presented to our obesity clinic with class 2 obesity and metabolic complications including prediabetes, dyslipidemia, and suspected obstructive sleep apnea (OSA). Anthropometric measurements revealed height of 170 cm, weight of 115 kg, and body mass index (BMI) of 39.8 kg/m^2^ (Class 2 obesity). Waist circumference was 120 cm, indicative of significant central adiposity (≥94 cm in men; International Diabetes Federation criteria). The patient reported marked hyperphagia with persistent hunger that exceeded the prescribed caloric intake, as documented by food diary review. His partner noted loud snoring at night, raising suspicion for OSA. He described himself as sedentary. He had no history of tobacco use and no family history of diabetes mellitus. No concomitant medications were being taken at the time of presentation, and no known drug interactions with incretin-based therapies were identified.

## Diagnostic assessment

Laboratory evaluation revealed impaired fasting glucose (IFG; fasting plasma glucose 110 mg/dL [6.1 mmol/L]; reference range: 70-99 mg/dL [3.9-5.5 mmol/L]) and hemoglobin A1c (HbA1c) of 5.8% (reference range: <5.7%), consistent with prediabetes. Fasting insulin was elevated, yielding a homeostatic model assessment of insulin resistance (HOMA-IR) of 5.0 (reference range: <2.0), placing the patient in the moderate-to-severe insulin resistance category [3]. Lipid profile showed borderline elevated triglycerides (165 mg/dL [1.86 mmol/L]; reference range: <150 mg/dL [<1.70 mmol/L]) and low HDL cholesterol (38 mg/dL [0.98 mmol/L]; reference range: ≥40 mg/dL [≥1.03 mmol/L] in men). Liver function tests and pancreatic enzymes (amylase and lipase) were within normal limits at baseline. Abdominal ultrasonography showed no cholelithiasis.

The patient's metabolic profile was consistent with an insulin-resistant obesity phenotype without diabetes, characterized by: central adiposity (waist circumference 120 cm), prediabetic glycemia (IFG, HbA1c 5.8%), moderate-to-severe insulin resistance (HOMA-IR 5.0), dyslipidemia (elevated triglycerides, low HDL), and marked hyperphagia. No criteria for type 2 diabetes mellitus were met. Pancreatic pathology was excluded by normal enzyme levels and imaging. The clinical picture was consistent with metabolic syndrome with preserved beta-cell function but significant peripheral insulin resistance.

## Treatment

Given the availability of 2 pharmacological options with distinct receptor profiles—tirzepatide (dual GIP/GLP-1 agonist) and semaglutide (selective GLP-1 agonist)—tirzepatide was selected as first-line pharmacotherapy based on its superior average weight loss outcomes in clinical trials [1, 2]. A hypocaloric diet was prescribed concurrently (1937 kcal/day, calculated using the Mifflin–St Jeor equation at 80% of the estimated total daily energy expenditure of 2421 kcal/day), along with structured lifestyle counseling. Dietary adherence was monitored throughout both treatment phases via regular food diary reviews. Nutritional counseling and encouragement of progressive physical activity were maintained during both phases.

During the first treatment phase (months 1-6), tirzepatide was initiated at 2.5 mg subcutaneously once weekly and escalated to the maximum approved dose of 15 mg weekly according to standard titration protocol. Prior to initiation, correct subcutaneous self-injection technique was verified with the patient using the tirzepatide autoinjector device (KwikPen®), and the patient demonstrated proficiency. The patient maintained full adherence, confirmed by pharmacy refill records and injection site examination. The prescribed hypocaloric diet (1937 kcal/day) was maintained throughout, verified by regular food diary reviews.

After 6 months at maximum dose, the patient achieved only 5.5 kg weight loss (4.8% of baseline body weight), corresponding to a post-tirzepatide weight of 109.5 kg (BMI ∼37.9 kg/m^2^), with persistent hyperphagia and no meaningful improvement in appetite control. Early response at 8 weeks was 2.1% weight loss, below the 5% threshold predictive of long-term success [4]. Waist circumference showed minimal change throughout this phase. No clinically meaningful improvement was observed in fasting glucose, HbA1c, HOMA-IR, or lipid parameters during tirzepatide treatment. Snoring and presumed OSA symptoms were unchanged. Pancreatic enzymes remained normal throughout; repeat measurement was performed per institutional monitoring protocol for GLP-1 receptor agonist therapy and in view of the patient's obesity-associated cholelithiasis risk. No gallstones were detected on repeat ultrasonography, which was performed for the same reason.

During the second treatment phase (months 7-12), following the assessment of inadequate response, tirzepatide was discontinued and semaglutide 2.4 mg subcutaneously once weekly was initiated after a 2-week washout period, using standard dose titration (starting at 0.25 mg weekly). Semaglutide (Wegovy®) is administered using a prefilled pen injector (Wegovy®), which differs from the tirzepatide device in design; the patient received device-specific training and again demonstrated correct technique prior to initiation. The same hypocaloric diet target and lifestyle recommendations were maintained. Food diary review during the semaglutide phase documented a spontaneous reduction in caloric intake to approximately 1650 kcal/day, below the prescribed 1937 kcal/day target, reflecting the pronounced appetite-suppressive effect of semaglutide.

## Outcome and follow-up

Within the first 4 weeks of semaglutide initiation, the patient reported marked reduction in hunger and early satiety. At 6 months, the patient had achieved a total weight loss of 23 kg (20% of baseline body weight), with a new weight of 92 kg and BMI of 31.8 kg/m^2^. Waist circumference decreased from 120 to 98 cm (a reduction of 22 cm). Fasting plasma glucose normalized to 88 mg/dL (4.9 mmol/L) from 110 mg/dL, and HbA1c decreased from 5.8% to 5.2%, representing resolution of prediabetes. HOMA-IR fell from 5.0 to 2.1, indicating resolution of insulin resistance. Triglycerides decreased from 165 mg/dL (1.86 mmol/L) to 110 mg/dL (1.24 mmol/L), and HDL cholesterol increased from 38 mg/dL (0.98 mmol/L) to 48 mg/dL (1.24 mmol/L).

Clinical improvements included complete resolution of hyperphagia, marked improvement in satiety, reduction in snoring intensity, and improved energy levels. No serious adverse events occurred. Mild transient nausea during dose escalation resolved spontaneously. Pancreatic enzymes remained normal throughout the semaglutide phase; repeat measurement was performed per institutional protocol for ongoing GLP-1 receptor agonist therapy. Repeat abdominal ultrasonography confirmed the continued absence of cholelithiasis, consistent with the original rationale for surveillance given obesity-associated gallstone risk. A comparison of outcomes across baseline, tirzepatide phase, and semaglutide phase is presented in Table 1.

**Table 1:** Comparison of clinical and metabolic parameters across treatment phases

Parameter	Baseline	After tirzepatide (Month 6)	After semaglutide (Month 12)
Body weight (kg)	115 kg	109.5 kg	92 kg
BMI (kg/m^2^)	39.8 kg/m^2^	37.9 kg/m^2^	31.8 kg/m^2^
Waist circumference (cm)	120 cm	∼120 cm	98 cm
Fasting glucose (mg/dL)	110 mg/dL	∼109 mg/dL	88 mg/dL
HbA1c (%)	5.8%	5.8%	5.2%
HOMA-IR	5.0	∼5.0	2.1
Triglycerides (mg/dL)	165 mg/dL	∼163 mg/dL	110 mg/dL
HDL cholesterol (mg/dL)	38 mg/dL	38 mg/dL	48 mg/dL
Caloric intake (kcal/day)	∼2421 (estimated TDEE)	1937 (prescribed diet maintained)	∼1650 (spontaneous reduction)
Weight loss (%)	—	4.8%	20.0%
Weight loss (kg)	—	5.5 kg	23 kg

Abbreviations: BMI, body mass index; HbA1c, hemoglobin A1c; HDL, high-density lipoprotein; HOMA-IR, homeostatic model assessment of insulin resistance; TDEE, total daily energy expenditure.

The long-term management plan includes continuation of semaglutide 2.4 mg weekly, maintenance of the hypocaloric diet with ongoing dietary counseling, gradual introduction of a structured physical activity program, polysomnography to objectively assess OSA, and regular monitoring of weight, metabolic parameters, and pancreatic enzymes. Weight management goals have been individualized based on the patient's metabolic response, risk reduction targets, and functional improvement, rather than a fixed BMI threshold.

## Discussion

This case presents a clinically important paradox: a patient with class 2 obesity achieved minimal weight loss with tirzepatide—a dual GIP/GLP-1 receptor agonist with superior average efficacy in clinical trials—but achieved 20% weight loss with semaglutide, a selective GLP-1 receptor agonist. Several nonmutually exclusive mechanisms may speculatively explain this differential response.

One possible explanation involves GIP receptor dynamics in states of marked insulin resistance. The GIP receptor (GIPR) is predominantly expressed in adipose tissue, where it regulates lipid metabolism and insulin sensitivity [5]. In states of marked visceral obesity and insulin resistance, GIPR expression is markedly downregulated, and residual GIPR signaling may paradoxically promote lipid storage [6]. Our patient's HOMA-IR of 5.0 places him in the moderate-to-severe insulin resistance category. It is speculative, however, to conclude that this HOMA-IR value alone constitutes sufficient evidence of GIPR dysfunction; tirzepatide achieves meaningful weight loss in many patients with comparable degrees of insulin resistance [7], and the mechanistic basis for this patient's nonresponse remains uncertain. In this context, tirzepatide's GIP component may not only have provided no incremental benefit over GLP-1 agonism alone, but could potentially have attenuated the appetite-suppressive effects of its GLP-1 component through competitive receptor interactions [7].

A second speculative mechanism involves intact central GLP-1 receptor signaling. GLP-1 receptors are highly expressed in key hypothalamic appetite-regulating nuclei (arcuate nucleus, paraventricular nucleus) and in the brainstem nucleus tractus solitarius [8]. Semaglutide's potent and selective GLP-1 receptor agonism directly activates these central pathways. The patient's dramatic appetite suppression within weeks of initiating semaglutide is consistent with, though not proof of, intact and highly responsive central GLP-1 receptor signaling that was apparently insufficient to overcome possible GIP-related interference during tirzepatide treatment.

Genetic factors may also play a role. Genetic variants in GIPR (eg, rs10423928) can reduce cAMP signaling and modulate the incretin effect [9]. Individuals with GIPR variants associated with reduced receptor function may derive minimal benefit from the GIP component of dual agonists, effectively rendering tirzepatide equivalent to or less effective than a selective GLP-1 agonist. Although genetic testing was not performed in this patient, his clinical phenotype is consistent with functional GIPR impairment, whether acquired through insulin resistance or genetically determined. This remains unconfirmed.

Recent evidence has identified severe insulin resistance (HOMA-IR ≥4-5), nondiabetic glycemic status, central obesity, and persistent hyperphagia as potential risk factors for tirzepatide nonresponse [3]. Conversely, absence of diabetes, high baseline BMI, and intact central GLP-1 signaling have been associated with favorable semaglutide response [10]. However, these associations are not established as sufficient criteria for treatment selection, and data from large prospective studies are lacking. Early weight loss at 8 weeks (<5%) is a validated predictor of long-term tirzepatide failure [4], and this patient's 2.1% early response could have prompted earlier therapeutic reassessment. The optimal timing of therapeutic switch—whether at 4 weeks, 8 weeks, or after full dose escalation—remains uncertain. Some patients may exhibit delayed responses to tirzepatide, and no prospective data currently define the ideal switchover window.

This case has several important limitations. GIP and GLP-1 circulating levels were not measured, precluding direct assessment of incretin axes. No polysomnography was performed to formally confirm OSA. No adipose tissue biopsy was obtained for GIPR expression quantification, and no genetic testing was conducted. The absence of these data substantially limits mechanistic certainty. The sequence effect (tirzepatide followed by semaglutide) cannot be entirely excluded, although the 2-week washout and identical dietary conditions across both phases minimize this concern. As a single case report, no generalizable conclusions regarding treatment algorithms or patient selection criteria can be drawn; prospective studies are required to validate whether pretreatment phenotyping can guide differential pharmacotherapy selection in obesity.

## Learning points

- Markedly elevated baseline insulin resistance (HOMA-IR ≥4-5) in a patient without diabetes may be associated with poor response to tirzepatide, potentially due to adipose tissue GIP receptor downregulation; however, this observation is based on a single case and requires prospective validation before informing treatment selection.
- Persistent hyperphagia despite dose escalation of tirzepatide is an early clinical warning sign of inadequate central appetite suppression and predicts poor long-term weight loss response.
- Early weight loss at 8 weeks (<5% body weight) is a validated predictor of tirzepatide nonresponse and should trigger timely therapeutic reassessment rather than prolonged continuation of ineffective therapy; the optimal timing of a therapeutic switch warrants further investigation.
- Nonresponse to tirzepatide does not predict nonresponse to semaglutide; tirzepatide nonresponders should be offered a therapeutic switch to semaglutide, as they may achieve excellent outcomes.
- Pretreatment phenotyping—including HOMA-IR, glycemic status, body composition, and appetite assessment—may inform personalized selection between GLP-1 and dual GIP/GLP-1 receptor agonists in obesity management, though prospective validation in appropriately powered studies is required before this approach can be formally recommended.

## Data Availability

Original data generated and analyzed during this study are included in this published article.
